# Bibliography

**Published:** 1898-09

**Authors:** 


### Bibliography.

Oral Pathology and Practice. A Text-Book for the Use of Students in Dental Colleges and a Hand-Book for Dental Practitioners. By W. C. Barrett, M.D., D.D.S., M.D.S., Professor of Oral Pathology in the University of Buffalo, Medical Department, etc. Published by the S. S. White Dental Manufacturing Company, 1898.

This volume, prepared by one of the best-known and clearest thinkers in the dental profession of the United States, will command wide attention. Whether this will be in agreement or otherwise, the book will fill a place among the text-books of college work. It is true, as the author states in his preface, that books upon dental pathology issued prior to the present year were not well adapted for "students in colleges as every day manuals for those who sought help in the hourly recurring complications of office life, they were two voluminous." In some instances, it may be added, they were too limited in scope. The author has aimed to strike a medium between these two extremes, and furnish the student with a compact volume of moderate price and not difficult to handle.

In view of this intention of the author, the book cannot be reviewed with the same attention to details as would be necessary in a work of greater magnitude. This volume, including index, makes a book of 239 pages.

The one peculiarity of this book, in which it differs from all others and which gives it a unique character, is the typography in which the prominent idea is sought to be conveyed. This is attempted to be impressed forcibly upon the mind of the student by heavy-faced type at the beginning of each distinct paragraph. This, while valuable to the untrained mind, becomes rather trying to one familiar with the subject, and it may be questioned whether it will aid materially the individuals for whom it is especially intended.

The author includes in his fifteen chapters some pathological conditions not usually incorporated in books upon dental pathology, such as pharyngitis, tonsillitis, diseases of the tongue, etc., entertaining the idea of "making of oral practice a true specialty of medicine." While this effort to broaden dental thought and practice has much to commend it, there still remains the question whether it is proper to transcend certain boundary lines and invade those of other specialties.

Chapters one to ten cover much valuable material, prepared with care, and briefly but thoroughly summarizing all the topics included under bacteriology: classification, fermentation, bacteriological pathology, septic and aseptic conditions, and inflammation, the latter covering five chapters.

The author's idea of proper teaching, as laid down under inflammation, is unquestionably the true one, that "it is infinitely better that the student in college should be given but one hypothesis rather than a number of conflicting theories," but as long as we have many men of many minds, this much-desired condition can never be reached. It is, however, one of the most troublesome features in teaching a professional subject. The writer has always contended that, upon practical lines, one system should alone be taught, but this fails the moment it comes in contact with the diversity of opinions in teaching force.

The chapter on "Diseases of Dentition" contains some very peculiar ideas, coming, as they do, from a dental practitioner. The prominent thought is expressed in the opening paragraph, "That it is possible for a retarded or disturbed dental development to induce very serious derangement is indisputable, but that it is a principal factor in inducing the great number of deaths that occur in children can scarcely be maintained. There are many cogent reasons for the contrary belief, while there is nothing, save the mere fact of coincidence, to sustain the theory too commonly accepted without inquiry or consideration." The author then follows with extended statistics of mortality in various foreign countries, and concludes that "nowhere is the cutting of teeth statistically given as the direct cause of mortality. Although it may in some instances induce death through some other complication, its influence is too insignificant to be included as a separate cause." It is surprising that the author should be willing to accept these statistics upon their face value when he must know that they are misleading, for it is a well-known fact that outside of dentistry dentition is not generally recognized as a possibly distinct pathological condition, in fact, is entirely set aside as a prominent factor in diseases of childhood by all except the most advanced thinkers in medical circles. Dentists who have accepted the opposite view through "inquiry and consideration" and, it may be added, extended experience, will regard the position taken by the author with regret. That his conclusions are wrong from the stand-point of the reviewer is certain, and they are certainly out of harmony with the ideas entertained by the leading thought of the dental profession.

It is not surprising, therefore, to find in the chapter upon the "Treatment of the so-called Diseases of Dentition," some very peculiar advice, especially in that where the appearance of the gum is to be the indicator for the use of the lance. While this is frequently a very sure guide, it is equally certain that reflex disturbance will occur at very early stages of development through pressure upon the pulps at the undeveloped portion of the roots, and this without any external evidence.

The reviewer has failed to find a single line upon the possible lesions of the permanent dentition. This is, to his mind, an extraordinary omission, when it is recognized as productive of some of the most serious pathological conditions. The first, second, and third molars have each their peculiar difficulties. The second especially through its development in the ramus of the inferior maxilla, and the third with its serious complications during the process of eruption.

The two chapters devoted to the pulp will, it is presumed, not meet the views of the large majority. The subject is practically limited to pulpitis and its treatment, which is too narrow even for the limitations the author placed upon his pen. The practical student will search in vain for any advice upon superficial pulpitis, pulp gangrene, pulp mummification or antiseptic precautions relating thereto.

The average dentist will be rather startled at this advice given under the treatment of alveolar abscess. "Warm fomentations may be used, a cloth rung out in hot water being applied to the face over the seat of trouble, and carefully covered, while the patient is kept warm." This certainly antagonizes all the teaching in this direction since the reviewer entered the dental profession. The fact that warm applications, at the place indicated, tend to produce a tissue of lessened resistance and invites the production of an external fistula seems conclusive to the writer. The advice given is not only contrary to established usage, but is wrong in fact, and will certainly lead the untrained into a maze of difficulties.

In considering "Deposits upon the Teeth," the author says that "the so-called 'green stain' of childhood is wholly superficial. It is called 'green stain,' although it may be dark or bronze or yellow in color." The colors here are somewhat mixed, for green stain has never before been combined with the yellow deposits. He further states that "it has then no special pathological signification, except so far as it may be a symptom of some unhealthy condition of the fluids of the oral cavity." That it leaves the enamel "uneroded" is not the experience of the writer.

The chapter on "Pyorrhœa Alveolaris," and especially the treatment, is not, in the reviewer's estimation, very satisfactory, and the author's statement that "very little dependence can be placed upon the many specific methods and remedies offered by those who claim to cure the incurable," must be regarded as unwarranted in view of the many positive statements made by individuals thoroughly versed in theory and practice. That pyorrhœa can be permanently cured admits of no question, and to assert that it is "incurable" is simply denying well-attested facts.

The author apparently has but little faith in cataphoresis in the treatment of hypersensitivity of dentine, for he says, "While, therefore, every progressive operator should use it, it is not now to be considered a finality. Its application must be simplified and its effects made positive by further experimentation before it can be so accepted."

The author ascribes the formation of nodular deposits to odontoblasts, for he says, "The former (odontoblasts) may exist enveloped in the pulp-tissue, and under the special stimulus that was perhaps responsible for their formation may commence functional activity, with the consequent organization of segregated spicules of dentine, and these may continue to grow until they assume the form of the usual pulp nodule." Upon what ground this hypothesis is entertained does not appear, and as it is not in accord with the generally accepted view of the origin of these calcific deposits some reason should have been given for the statement. A wide distinction must be made between secondary dentine and the deposits in the pulp. The odontoblastic layer has all to do with the development of the former, but, apparently, has no relation with the latter.

It is unnecessary to follow the author through the remaining chapters. They as well as all those preceding are written with the author's usual ability and terseness of style.

The differences which naturally arise between reviewer and author are more the result of different experiences, and do not necessarily involve the general character of the book. This one will occupy a place in the text-book series not exactly filled by any previous work. The idea generally entertained of a text-book is that it must be absolutely in accord with accepted opinions. This, in the writer's opinion, is an error, for the fact that the volume presents the personal views of the author gives it a special value not to be accorded to a book prepared through compilation. It undoubtedly will find a place among the recognized works used in dental college instruction.

Cataphoresis or Electric Medicamental Diffusion as applied in Medicine, Surgery, and Dentistry. By William James Morton, M.D., Professor of Diseases of the Mind and Nervous System and Electro-Therapeutics in the New York Post-Graduate Medical School and Hospital, etc. New York: American Technical Book Co., 45 Vesey Street, 1898.

The author says of this book that "this treatise has been written at the request of some of my friends, earnest practitioners in medicine and dentistry, particularly the latter profession, who desire information, not alone in regard to the mere practice of the art of electric diffusion, but also in the fundamental principles which govern this art."

Cataphoresis has had such a rapid development that it is not surprising to find the able author and one of the practical originators giving to the medical and particularly the dental profession a work which will remain as an authority upon this subject.

The author begins his book with the history of the subject,—a valuable contribution to our knowledge in this direction. In the chapter upon its "Developments in Dental Surgery" the author does full justice to the earlier experimentation, and will thus set at rest disputes in regard to priority.

The "Writer's Contributions" constitutes an interesting chapter, and exhibits a thoroughness in every step taken worthy the highest commendation. As an illustration of this the following quotation is given, in which he attempted to prove the ease with which "solid particles might be driven into tissue by the action of the electric current. Some finely powdered lamp-black or graphite was incorporated with some salicylate of soda and placed on my arm under the positive electrode. The current was turned on and the particles of graphite were carried into the sweat-follicles, making small black spots, like bird-shot, which were so deeply embedded that they did not disappear for several weeks."

The chapter on "Elementary Electrical Principles" will be of great value to those who are beginning the practice of cataphoresis without the necessary knowledge of electricity. The elementary principles are so clearly elucidated that the unlearned will find the subject so simplified as to be easy of comprehension. The importance of this cannot be over-estimated, for many have rushed into the use of this method without any definite idea of the meaning of the words volt, ohm, ampère, coulomb, watt, or joule, or of the possible injurious effect which may be produced through ignorance.

The chapters on electrodes, both medical and dental, are fully illustrated and very suggestive.

Upon the "Anæsthetization of Sensitive Dentine" the author is careful in his directions in order to avoid accidents, and directs "not to break the circuit . . . by removing the electrode from the tooth with the current turned on, . . . and not to leave the patient during the administration, as an accident may occur by the patient interfering with the electrodes. . . . It is well to insulate the chair by placing linoleum or rubber under its feet." It is doubtful whether these precautions are taken by the majority of operators.

The author maintains that "the best results are to be obtained either from a strong solution of hydrochlorate of cocaine or a solution of hydrochlorate of cocaine with guaiacol, which I was the first to propose. . . . My preference is for the guaiacol-cocaine, principally because of its greater rapidity of action, its sterilizing effect, and its reduction of the chances of toxic effects from cocaine by preventing its diffusion to the general circulation."

The chapter on "Bleaching of Teeth by Cataphoresis" is interesting and practical, and is fully illustrated by experiments out of the mouth as well as by cases in actual practice. The value of this process is now so generally recognized that, whatever may be the final outcome of cataphoresis, this part of it, without doubt, will remain as a permanent method of practice.

While it is not possible as yet to determine the full value of this treatment in its relation to sensitive dentine and to the pulp, sufficient has been proved to demonstrate its importance, and those who make use of it should be thoroughly informed in the *minutiæ* of procedures necessary to produce satisfactory results. The fear, not by any means exaggerated, that eventually disastrous results would follow the use of this process has thus far not been confirmed. It is true that the few cases reported of death of the pulp tend to enforce the necessity of caution and greater knowledge of the agents used. There is evidently less enthusiasm regarding cataphoresis than was manifested at its earlier introduction, but that it will become a useful aid in the practice of certain lines of dentistry cannot, with our present experience, be disputed, and the practitioner will not require a better or safer guide than this volume of two hundred and sixty-seven pages. It should become a text-book upon this subject in all the dental colleges of the country, for the demands of the future will require thorough teaching in this direction. For the practitioner it cannot fail to be of constant value in his daily operations.

